# Degradation of Temminck's pangolin (*Smutsia temminckii*) scales with a keratinase for extraction of reproductive steroid hormones

**DOI:** 10.1016/j.mex.2021.101229

**Published:** 2021-01-13

**Authors:** Arantxa S. Blecher, Juan Scheun, André Ganswindt

**Affiliations:** a Mammal Research Institute, Department of Zoology and Entomology, Faculty of Natural and Agricultural Sciences, University of Pretoria, South Africa; b Biodiversity Research Services, National Zoological Gardens, South Africa; c Department of Life and Consumer Sciences, University of South Africa, South Africa

**Keywords:** Pangolin scales, Keratinase, Reproduction, Progestagens, Androgens

## Abstract

Hormone monitoring in keratinous tissues has become increasingly popular. The insoluble keratin materials are generally pulverised before hormone extraction; however, this is difficult for thicker keratin structures like baleen plates or hooves. A new method, involving the use of keratinase, allows enzymatic digestion of keratin and hormone analysis in the resulting suspension. Pangolins are unique mammals covered in keratinous scales, which are one of the reasons these animals are extensively trafficked. This study aimed to investigate the suitability of Temminck's pangolin scales as hormone matrix for quantifying reproductive steroids. A protocol was developed to digest scales with a keratinase before measuring hormone concentrations. This method can be used to investigate the reproductive endocrinology of Temminck's pangolins but may also be extended to the other extant pangolin species.

• Keratinase digests Temminck's pangolin scales and reproductive steroid metabolite concentrations are measurable in the resulting suspension.
• Isopropanol is an ideal washing solvent for scales to remove surface contaminants and scale sample mass should be standardised to allow comparisons.
• Any section of a scale and scales from any pangolin body region can be used as samples for hormone quantification.

## Graphical abstract

**Figure:**
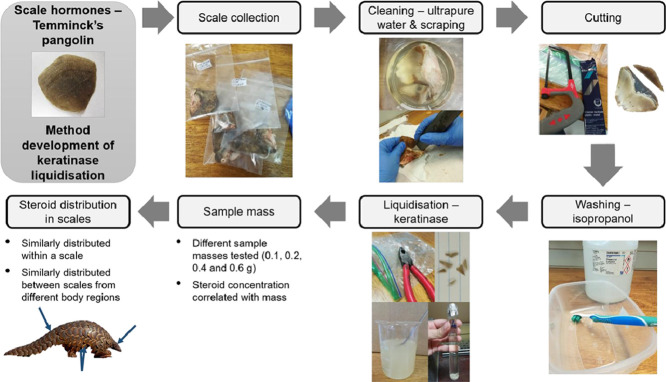

Specifications table

**Table:** 

Subject area:	Agricultural and Biological Sciences
More specific subject area:	*Zoology*
Method name:	*Using a keratinase for improved extraction of hormones from keratinous tissue*
Name and reference of original method:	Alba, A.C., Strauch, T.A., Keisler, D.H., Wells, K.D., Kesler, D.C., 2019. Using a keratinase to degrade chicken feathers for improved extraction of glucocorticoids. General and Comparative Endocrinology 270, 35-40.
Resource availability:	Keratinase enzyme Cibenza IND900 (Novus International, Incorporated., St. Charles, Missouri) https://www.novusint.com/Products/cibenza#fndtn-cibenzaind900

## Method details

### Background information

Hormone monitoring is useful for evaluating the responsiveness of the reproductive and stress axes to environmental and social factors driving animal health and reproductive success [1,2]. Circulating hormone concentrations can be measured directly in plasma and serum, while hormone metabolites are present and measurable in urine and faecal samples [3], [4], [5], [6]. In addition to excreta and blood, alternative hormone matrices include saliva, milk, eggs, hair, and feathers [3,7]. Hair and feathers, along with other epidermal appendages like nails, claws, scales, beaks, and baleen, are made up of keratin [8]. This naturally insoluble material, known for its durability [8,9], has been used as hormone matrix. Popular choices are hair [e.g. 10,[11], [12], [13]] and feathers [14,15], while other examples include whale baleen [16,17], claws [18], snakeskin [19] and reptilian scutes [20].

In preparation for hormone extraction, most keratinous materials are pulverised in various ways to convert them into a suitable form for extraction [e.g. 12,16,18]. Alternatively, Alba et al. [21] used a keratinase for improved hormone extraction from chicken feathers. Keratinases are enzymes in the family of serine endoproteases produced by microorganisms and are capable of degrading insoluble keratin substrates [22], [23], [24], [25]. So far, research on keratinases has mainly focussed on their functions in the feed and fertiliser industry, detergent production, the leather industry and biomedical applications such as degradation of prion proteins [23,26]. Only Alba et al. [21] have used the enzyme for optimising hormone extraction from a keratin material. In their study, the chicken feathers were not ground into a powder but liquidised in a keratinase solution, and the resulting suspension was successfully used to measure corticosterone metabolite concentration [21]. These results suggest that other keratin-based materials like nails, hooves, and scales, can be degraded using keratinase, though further research is required to confirm this.

Scales are mainly found in reptiles and birds, but pangolins are unique mammals in that most of their skin is covered in keratinous scales that make up almost 20% of their bodyweight [27]. The scales are similar to primate nails and whale baleen plates and are made up of both α- and β-keratin types [28], [29], [30], [31]. Therefore, a keratinase may degrade pangolin scales to determine hormone concentrations in the resulting suspension. The present study aimed to adapt the method described by Alba et al. [21] for use on Temminck's pangolin (*Smutsia temminckii*) scales, to evaluate the ability to monitor reproductive hormone metabolite concentrations in such scales.

### Scale sampling

Temminck's pangolin scales used in this study were obtained from previously confiscated batches or removed from pangolin carcasses; both of which were stored at a secure facility in South Africa. Where possible, sex (morphologically) and approximate age class (juvenile, sub-adult, or adult based on body size) of the pangolin carcasses were identified after confiscation. Scales were removed from the carcasses using razor and scalpel blades, ensuring that any residual flesh and skin was scraped off as thoroughly as possible. These samples were stored at -20 °C to avoid decay of flesh remaining on the scales. In contrast, loose scales from collection bags were already rid of flesh and skin, and could thus be stored in plastic bags at room temperature. The study was approved by the University of Pretoria Animal Ethics Committee (NAS078/2019) and the SANBI Research Ethics and Scientific Committee (NZG/RES/P18/06).

### Initial cleaning of scales

To clean the scales of any residual flesh and skin completely, two techniques were tested. One set of scales was freeze-dried for three days directly after removal from the carcass to dry out the tissue still present on the scales. It was then attempted to scrape off the dried tissue with small knives, however, this proved ineffective as the dried flesh was firmly attached to the scale and difficult to remove. The second set of scales was submerged in ultrapure water and left to soak overnight. This softened the tissue on the scales and it could be easily removed by hand or with scalpel blades. Moreover, other materials, such as blood and sand on the scales, were also washed off in the water. Therefore, submersion in ultrapure water was an effective method for removal of residual tissue from pangolin scales, a suitable method also applied to whale baleen plates [17]. In the present study, all scales that had been soaked in ultrapure water were freeze-dried for three days after cleaning to allow comparison to the first set of scales that were only freeze-dried. Further investigation is needed whether drying after submersion is necessary, however, Fernández Ajó et al. [17] did not include a drying step and it could thus be excluded here in future experiments.

### Cutting of scales

Scales were fastened in a clamp between two sponges and cut into desired pieces using a small hacksaw (MITCO Junior Hacksaw, blade length = 153 mm). The small hacksaw was found most effective for cutting scales, as it allowed precision of movement and minimised splintering of the scale. To further avoid splintering at the scale edges, a small incision into one edge of the scale was made by moving the saw in one direction or by holding the piece being cut off by hand. The thicker parts of the scale could be cut using normal sawing motion.

**Fig. 1:**
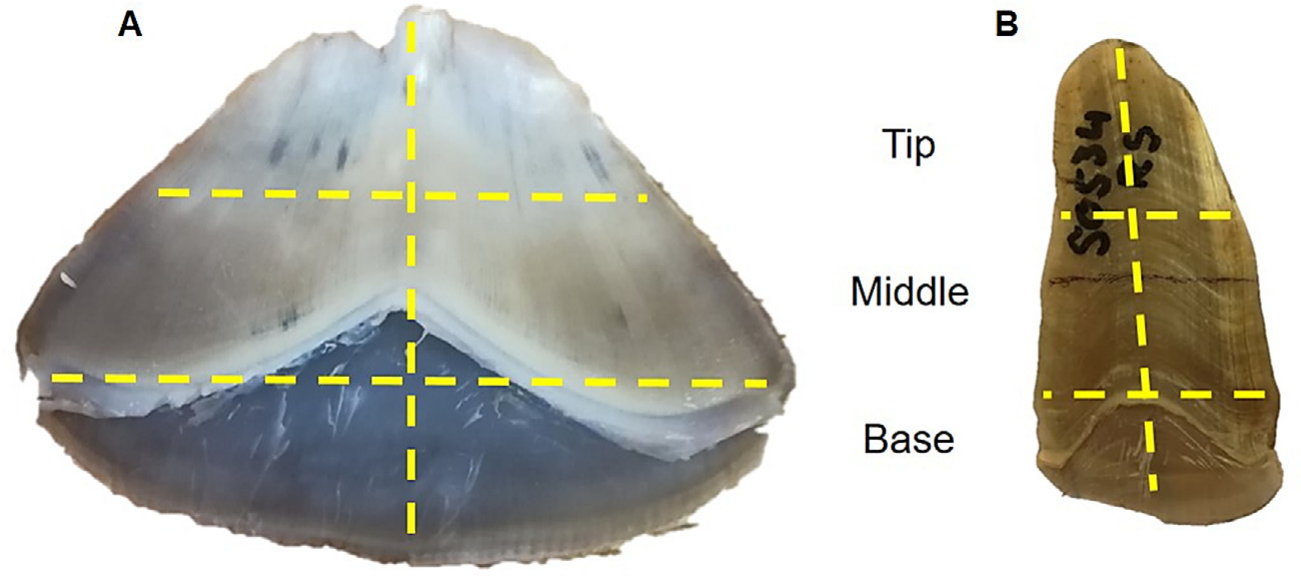
Two Temminck's pangolin scales from different body parts, showing the variation in shapes of pangolin scales. Scale A represents a large and broad scale mainly found on the back and tail, while scale B is a smaller and thinner scale found on the limbs and sides of the body. Dashed lines on each scale show how scales were divided into base, middle and tip sections, as well as halved along the vertical middle line from base to tip.

In the present study, pangolin scales were divided into three sections: base, middle and tip, and depending on the experiment, different sections were selected. The base of the scale consisted of the area where the scale was attached to the pangolin's epidermis, while the tip was the thinnest area at the exposed end of the scale and the middle section the thickest scale region (Fig. 1). The size and mass of a scale section used depended on the body location the original scale was taken from, where some scales are broader and thicker (Fig. 1A), while others are narrower and thinner (Fig. 1B).

### Further washing of scale samples

All scale samples had to be washed with a selected alcohol to remove surface contaminants, such as dried blood and human skin excretions, thus avoiding their inclusion in the final liquidisation suspension. As alcohols, like methanol, are used for hormone extraction from keratin tissues [e.g. 16,18,19], a suitable washing agent that would not lead to hormone extraction from the scales, had to be chosen. Isopropanol was previously suggested as a suitable washing agent for hair [11,12], thus it was chosen for testing on pangolin scales.

One scale was thoroughly washed under tap water with a brush to clean off visible dirt and blood. Next, the scale was cut into base, middle and tip sections, of which the base and middle sections were selected for this test due to appropriate overall mass. Each piece was separately placed into a tray filled with enough 100% isopropanol to cover the piece completely and brushed with a toothbrush for approximately 30 s. Pieces were then patted dry with paper towel and 1.5 ml of the wash solvent in the tray was collected as an initial sample. Next, the scale pieces were placed individually into beakers containing 20 ml of 100% isopropanol and set on a magnetic stirrer for 15 min. At 5 min intervals (5, 10, 15 min), 1.5 ml of the wash solvent were collected. After 15 min, the scale pieces were removed from the isopropanol and the collected wash solvent samples were stored frozen until hormone analysis.

Progestagen and androgen metabolite concentrations in the wash solvents were measured with relevant enzyme immunoassays (EIA; see *Enzyme immunoassays* section for details). Metabolite concentrations showed no increase, even after the scale pieces were washed for 15 min (Table 1). Therefore, isopropanol appears as a suitable wash solvent to remove surface contaminants on the scale, while having minimal draining effect on the hormone concentrations of the scale.

**Table 1:** Progestagen and androgen metabolite concentrations in wash solvent, measured with EIAs, following four consecutive washes of a Temminck's pangolin scale with 100% isopropanol. The scale was divided into base and middle sections, which were washed separately.

Scale section	Scale piece mass (g)	Washing time (min)	Progestagen metabolite concentration (ng/g scDW)	Androgen metabolite concentration (ng/g scDW)
base	0.45	0	13.51	4.22
		5	11.13	4.41
		10	9.35	2.36
		15	10.68	3.99
Middle	1.90	0	2.99	1.13
		5	3.22	0.82
		10	3.50	0.63
		15	3.20	0.97

A second washing experiment was conducted to investigate whether a hormone washout effect could be detected in scale samples after washing in isopropanol. For this, another scale was divided into half and then into base, middle and tip sections. The three pieces from one half were washed with isopropanol and a toothbrush for 30 s (short wash), while the pieces from the other half were submerged in isopropanol and washed on a magnetic stirrer for 15 min. Scales were then patted dry with paper towel and prepared for liquidisation (see section: *Liquidisation method of scale samples with keratinase*), after which hormone metabolite measurements took place. For each washing treatment, a mean of scale progestagen metabolite (scPM) and scale androgen metabolite (scAM) concentrations for the three scale sections was calculated and subsequently used to compute the percentage difference in hormone metabolite concentrations between the short wash and the 15 min wash. A minor to moderate increase in scale hormone concentrations correlated with washing time; of about 10% for scPM concentrations (short wash = 594.87 ng/g scDW; 15 min wash = 660.28 ng/g scDW), and 38% for scAM concentrations (short wash = 74.08 ng/g scDW; 15 min wash = 108.48 ng/g scDW). Consequently, a standard procedure of washing all scale samples with isopropanol and a toothbrush for approximately 30 s was adopted to remove surface contaminants from the scale for any further experiments.

### Liquidisation method of scale samples with keratinase

To prepare scale samples for hormone analysis, they were liquidised using the method first described by Alba et al. [21] for chicken feathers. Liquidisation was achieved using the keratinase enzyme Cibenza IND900 (Novus International, Inc., St. Charles, MO; EC 3.4.21; enzyme activity (min) = 65,000 U/g), which is sourced from *Bacillus licheniformis* and consists of *B. licheniformis* fermentation solubles and sodium sulphate. Before scales samples were liquidised, they were clipped into small pieces (approximately 5 mm by 7 mm) using linesman pliers. This was done inside a clear plastic bag to avoid scale pieces being lost during the clipping process.

For liquidisation, a keratinase solution as described by Alba et al. [21] was prepared by mixing the keratinase enzyme with phosphate buffered saline (PBS; pH = 9.0). Scale samples were then placed in test tubes with 15 ml of keratinase solution, and the top of the tube was covered with aluminium foil. Samples were incubated for four days in a water bath incubator (LABEX PTY LTD, JP SELECTA^Ⓡ^, UNITRONIC-OR shaking water bath) and stirred approximately every 24 h, either by plugging the top of the tube and inverting it once or stirring with a rod. Liquidisation was observed by the appearance of white powder-like material settling at the bottom of the test tubes. Incubation was terminated after four days when no further liquidisation was visibly detected, even though scale pieces were not completely degraded. An incubation time of four days was thus set for all experiments. Test tubes were then removed from the water bath, their entire contents emptied into 50 ml centrifuge tubes, and stored at -20 °C until hormone analysis.

Although the possibility of steroid conversion by the utilised keratinase enzyme cannot be completely excluded, it appears rather unlikely that the structure of the quantified androgens and progestagens would change during incubation. Keratinases specifically cleave disulphide bonds of keratin materials, opening the complex keratin structure [24,32], thus they are not likely to interact with the hormones and their metabolites in the suspension.

### Enzyme and temperature experiment

As scale pieces were initially not completely degraded, we evaluated whether the keratinase concentration (1 g enzyme in 30 ml PBS) or the incubation temperature (45 °C), proposed by Alba et al. [21] for feather liquidisation, was appropriate for pangolin scale liquidisation and whether an increase in one or the other parameter would facilitate complete scale sample degradation. The proposed concentration and incubation temperature were compared to a three-fold increase in keratinase concentration (3 g enzyme in 30 ml PBS) and a 7 °C increase in temperature (52 °C).

In this experiment, ten pangolin scales of unknown origin were cleaned under tap water using a brush, and four 0.40 g pieces were cut from each scale (from the base and middle sections). The 40 resulting pieces were then washed with isopropanol and clipped as explained above. The four pieces from a scale were each used in four different treatments: original and increased enzyme concentration (incubation temperature at original 45 °C), as well original and increased incubation temperature (enzyme concentration at original 1 g enzyme in 30 ml of PBS). To compare different enzyme concentrations, one piece from each of the ten scales was liquidised separately using a solution with the original enzyme concentration, while another ten pieces were liquidised using a solution with the increased enzyme concentration (3 g enzyme in 30 ml of PBS). The remaining 20 scale pieces were used similarly to compare the original and increased (52 °C) incubation temperature.

**Fig. 2:**
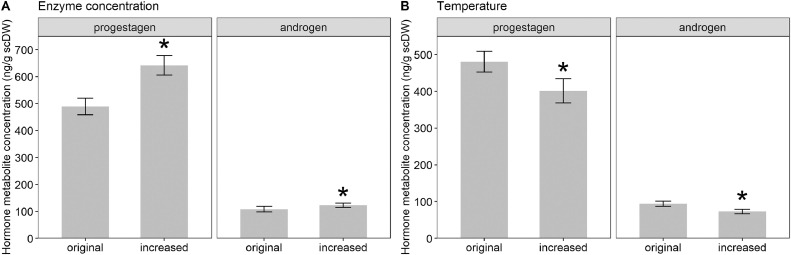
Progestagen and androgen metabolite concentration (ng/g scDW) determined with EIAs in Temminck's pangolin scales incubated at original (1g enzyme per 30ml phosphate buffered saline) and increased (3g enzyme per 30ml phosphate buffered saline) enzyme concentrations (A) or at original (45°C) and increased (52°C) temperatures (B). Temperature was kept constant at 45°C for the enzyme concentration comparison, while enzyme concentration was kept constant at 1g enzyme per 30ml phosphate buffered saline for the temperature comparison. Significant differences between data set pairs are indicated by *.

Respective scPM and scAM concentrations were measured in the resulting suspensions and the effect of increased enzyme concentration or incubation temperature was evaluated using paired t-tests. Both mean scPM and mean scAM concentration were significantly higher if scales were incubated in a solution of increased enzyme concentration (*t* = 7.20, df = 9, *p* < 0.01 and *t* = 3.23, df = 9, *p* < 0.05 respectively; Fig. 2A). When increased incubation temperature was compared to the original temperature, both scPM and scAM concentrations were significantly lower when incubated at increased temperatures (*t* = 4.16, df = 9, *p* < 0.01 and *t* = 8.31, df = 9, *p* < 0.01 respectively; Fig. 2B). Additionally, neither an increase in enzyme concentration nor an increase in incubation temperature achieved full degradation of scale samples.

Although scales were not fully degraded and incubation in a keratinase solution with a higher enzyme concentration yielded significantly higher hormone concentrations in the suspensions, scale samples incubated in solutions with a lower keratinase concentration also showed sufficient readouts. Less enzyme is needed for solutions of lower concentrations, thus making it more financially suitable. Therefore, a keratinase concentration of 1 g per 30 ml phosphate buffer appears suitable for pangolin scale degradation. Regarding incubation temperature, an increased incubation temperature decreased the final hormone concentration in the suspensions, therefore, incubation temperature was kept at 45 °C, as originally proposed by Alba et al. [21].

### Sample mass experiment

**Fig. 3:**
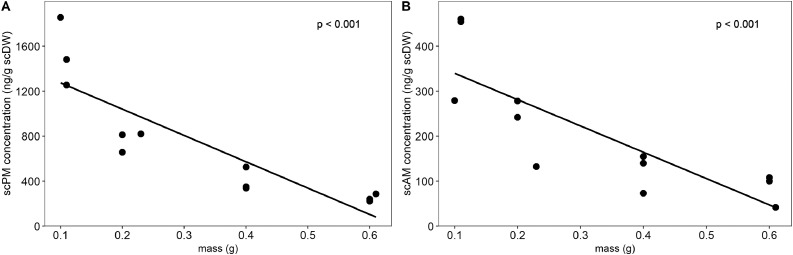
Progestagen (A) and androgen (B) metabolite concentration (ng/g scDW) in Temminck's pangolin scale pieces ranging from a mass of 0.1 g to 0.6 g. The p-values of the correlation tests are indicated.

For further optimisation of the steroid extraction process, a suitable sample mass for the scales had to be determined. To investigate the effect of varying sample mass on resulting hormone concentrations in scales, three scales (one from an adult female and two from adult males) from the tail of a pangolin were used. From each scale, the base section was cut off and further subdivided into samples with differing masses (low: 0.10–0.11 g; medium-low: 0.20–0.23 g; medium-high: 0.40 g; high: 0.60–0.61 g). Scales were cleaned with ultrapure water and isopropanol, liquidised, and analysed. Pearson correlation tests were performed to investigate whether scPM and scAM concentrations correlated with sample mass. The scPM concentration showed a significant negative correlation with sample mass (*R* = -0.87, *p* < 0.001), as did the scAM concentration (*R* = -0.83, *p* < 0.001), with highest hormone concentrations observed in samples with low mass and vice versa (Fig. 3).

Previous studies have shown a similar small sample artefact in faecal [33,34], hair [35] and feather samples [36], [37], [38]. Therefore, the amount of analysed scale material should be standardised across samples to allow a comparison of respective hormone values determined. Based on our findings, a sample mass of around 0.40 g appears sufficient in terms of obtainability from various scales, as well as subsequent hormone quantification and is thus suggested for experiments going forward.

### Scale part comparison

It was further evaluated whether hormone metabolite concentrations differ between scale sections. Six scales (three from females and three from males) were divided into base, middle and tip sections, and from each, a 0.40 g sample was cut, cleaned, and liquidised. Both scPM and scAM concentrations were compared between the three scale sections using a linear mixed-effects model with scale section as a fixed effect and individual ID as random effect.

**Fig. 4:**
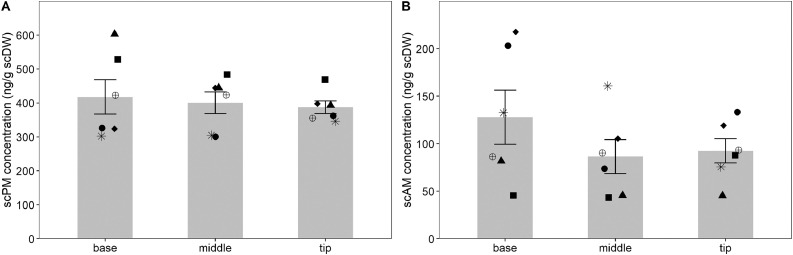
Scale progestagen (A) and androgen (B) metabolite concentration (ng/g scDW) in six Temminck's pangolin scales from three different scale sections. Individual data points are indicated by different symbols and scale sample mass was standardised at 0.40 g.

Respective scPM (*χ²* = 0.77, df = 2, *p* = 0.68) and scAM (*χ²* = 3.78, df = 2, *p* = 0.15) concentrations did not differ significantly between pangolin scale sections (Fig. 4). Previous studies on the distribution of hormone metabolites in keratinous matrices have mainly focused on cortisol or corticosterone analysis in hair and feathers. Similar to the results of the present study, several studies have shown that hormone metabolite concentration does not vary along consecutive hair segments [12,^39^,40]. However, other studies demonstrated significant differences in steroid concentrations between sections of hair and feathers [37,^41^,42]. Furthermore, steroid metabolite concentrations in keratin may not only show species but also individual variation. For example, steroid analysis in whiskers of different seal species showed that in some species, steroid metabolite concentrations differed between whisker sections, while in others they did not [43,44]. In chimpanzees, hair cortisol metabolite concentrations showed a high level of individual variation between the distal and proximal hair sections [35]. Therefore, the distribution of steroid hormone metabolites in keratinous materials seems highly variable.

While some studies suggest hair can be divided into sections to create a time scale of hormone deposition [45] or have successfully created time scales, for example in whale baleen [16,^17^,^46^,47] and feathers [14,^15^,48], it remains unclear how steroids are deposited into the tissue and whether it reflects a historical record or more recent events [see e.g. 49]. Evidence from bears, where increased cortisol in hair due to capture stress could be seen after only minutes, suggests that cortisol incorporation into hair is faster than originally thought [50]. Moreover, Colding-Jørgensen et al. [51] and Kalliokoski et al. [49] argue that steroids are not locked in place in hair, but rather diffuse through the hair. Therefore, at least hair is more likely a matrix for more recent rather prolonged steroid concentration [49,51]. However, as keratin tissues are very different in density and growth rate, comparisons between the different matrices are difficult and should be done with caution. For pangolin scales in particular, the growth rate is unknown and only few studies have investigated scale composition and growth [27,^28^,^30^,31]. It thus remains unknown how steroids are incorporated into the scale during its growth, however, results from the present study confirm that any part of a scale can be used for progestagen or androgen quantification.

### Body region comparison

We also evaluated whether scPM and scAM concentrations in scales differ between different body regions. For this experiment, four scales were collected from nine male (six adults, two sub-adults, and one juvenile) and five female pangolins (two adults, one sub-adult, and two juveniles). Scales were removed from four body regions: one from the top of the tail base (where the tail is attached to the body), one from the dorsal region of the head, and one from the right and left sides of the body (in the middle between the fore- and hind-legs). From each of the scales, the base section was cut off and a 0.40 g piece was used. Samples were cleaned and liquidised using the methods explained previously. To compare the four body regions, scPM and scAM concentrations of all age classes were grouped but analysed separately for each sex using linear mixed-effects models, with body region as a fixed effect and individual ID as random effect.

**Fig. 5:**
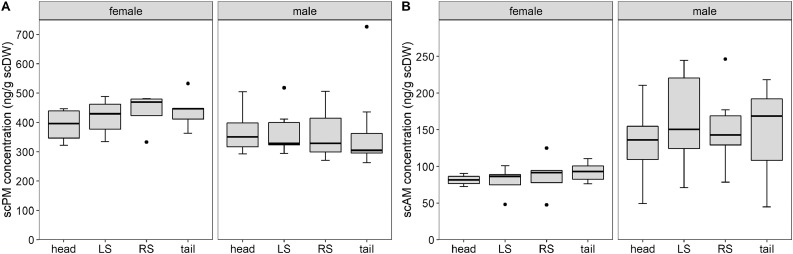
Progestagen (A) and androgen (B) metabolite concentration (ng/g scDW) in scales of male and female Temminck's pangolin at four different body regions: head, LS (left side), RS (right side) and tail. Only the base section of each scale was used and sample mass was standardised at 0.40 g.

Model results showed that scPM concentration was not significantly different between body parts of females (*χ²* = 2.97, df = 3, *p* = 0.40; Fig. 5A) or males (*χ²* = 0.83, df = 3, *p* = 0.84; Fig. 5A). Similarly, scAM concentration was not significantly different between body parts of females (*χ²* = 1.40, df = 3, *p* = 0.70; Fig. 5B) or males (*χ²* = 5.38, df = 3, *p* = 0.15; Fig. 5B). As with investigations on the distribution of hormone metabolites within a matrix, research on differences in hormone metabolite concentration between samples from different body regions has focused on cortisol or corticosterone in hair and feathers. An absence in region-specific differences in hormone metabolite concentration, as found in this study, was previously described for dog hair [52] and finch feathers [53]. In contrast, several studies have found differences in hormone metabolite concentrations between body regions [10,^38^,^54^,55]. These differences appear to be species-specific, where one species shows variation in hormone metabolite concentration between body regions, while a similar species may not, for example in grizzly and polar bears [40,56], as well as chimpanzees and orang-utans [39,41]. As there is no variation in scPM or scAM concentration in Temminck's pangolin scales from different body regions, this experiment showed that any Temminck's pangolin scale can be used for progestagen and androgen quantification.

### Enzyme immunoassays

EIAs described by Schwarzenberger et al. [57] and Palme and Möstl [58] were used to determine immunoreactive scPM and scAM concentrations in the suspensions, respectively. Full descriptions of the assay components including cross-reactivities are provided by the respective references listed above. EIAs were performed directly on the digested suspensions, using the process described by Ganswindt et al. [59]. In brief, serial dilutions of the standard stock and dilutions of antibody and biotin-labelled steroid were prepared using assay buffer. Then, 50 μl of either assay buffer, standards, quality controls (high and low) or digested sample suspension were added in duplicates to the wells of an lgG coated 96-well microtiter plate. Following this, 50 μl of biotin-labelled steroid solution and antibody solution were added to each well. Plates were then stored overnight at 4 °C for incubation. Subsequently, the liquid was discarded and the plate washed four times before 150 μl of streptavidin-POD solution was added to every well and the plate was incubated at 4 °C with gentle shaking for 45 min. After this, all liquid was discarded again and the plate washed four times and patted dry before 150 μl of substrate solution was added to every well. Then the plate was incubated at 4 °C with gentle shaking until the optical density of the highest concentrated standard reached ~1.0. The enzymatic reaction was terminated by adding 50 μl H_2_SO_4_ (2 M) to the wells. Finally, optical density was determined at 450 nm and 620 nm and results calculated using a best-fit curve.

Overall higher scPM values in females compared to males, as well as overall higher scAM values in males compared to females, indicate the detection of biological meaningful hormone values. Sensitivity for the progestagen EIA was recorded at 12 ng/g scale dry weight (scDW) and 1.50 ng/g scDW for the androgen assay. Intra-assay coefficients of variation (CV), determined by repeated measurements of high and low value quality controls, were 5.64 and 6.12% for the progestagen and 5.22 and 6.82% for the androgen EIA, while similarly determined inter-assay CVs were 12.33 and 14.19% for the progestagen and 11.27 and 13.14% for the androgen EIA. Serial dilutions of scale extracts gave displacement curves parallel to the respective standard curves, with the relative variation of the slope of the trendlines recorded at less than 2% for the androgen EIA and less than 1% for the progestagen EIA. All assays were performed as first described by Ganswindt et al. [59] at the Endocrine Research Laboratory, University of Pretoria, South Africa.

### Statistical analysis

All statistical analyses were done using RStudio [60]. Normality was tested with the Shapiro-Wilk normality test or visually determined using Q-Q plots, and homogeneity of variances was evaluated using Levene's test (R package ‘car’ [61]). Linear mixed-effects models were used from the R package ‘lme4’ [62]. Results are presented as mean ± standard error and *p*-values < 0.05 were regarded as significant.

## Conclusion and future recommendations

In conclusion, Temminck's pangolin scales appear as a suitable matrix for determining individual progestagen and androgen metabolite concentrations, and the use of keratinase is an ideal method for scale sample extraction prior to EIA analysis. The method developed in the present study can now be applied to investigate reproductive endocrinology in Temminck's pangolins, but may also be extended to the other extant pangolin species across the globe. This is particularly applicable as pangolin scales form a major part of the global trade in pangolins and are often the only available tissue confiscated from the trade. Additionally, scales are easily storable and present an accessible matrix for hormone analysis. We recommend further investigation into the growth of pangolin scales and how this may influence the deposition of hormone metabolites into the scales, as well as adaptation of this method for use on other keratinous materials such as hooves, antlers, claws, and horns from various taxa.

## Declaration of Competing Interest

The authors declare that they have no known competing financial interests or personal relationships that could have appeared to influence the work reported in this paper.
